# Repeated Unstimulated Whole Saliva Collection: A Reliable Approach to Improve Diagnostic Accuracy

**DOI:** 10.1111/joor.70172

**Published:** 2026-02-25

**Authors:** Gennaro Musella, Filippo Sisto, Maria Eleonora Bizzoca, Nicola Cirillo, Lorenzo Lo Muzio, Giuseppe Troiano, Rosa María López‐Pintor, Vito Carlo Alberto Caponio

**Affiliations:** ^1^ Department of Clinical and Experimental Medicine University of Foggia Foggia Italy; ^2^ Department of Medicine and Surgery University LUM Casamassima Italy; ^3^ ORALMED Research Group, Department of Dental Clinical Specialties, School of Dentistry Complutense University Madrid Spain; ^4^ Department of Life Sciences, Health and Health Professions Link Campus University Rome Italy

**Keywords:** diagnostic techniques and procedures, repeated measurements, saliva, sialorrhea, xerostomia

## Abstract

**Background:**

Unstimulated whole saliva (UWS) sialometry is fundamental for diagnosing salivary gland hypofunction, but single‐timepoint measurements are limited by physiological variability.

**Objective:**

To evaluate intraindividual variability and reproducibility of a repeated‐measures UWS collection protocol in healthy young adults, and to propose a standardised approach to improve diagnostic accuracy.

**Methods:**

Sixty‐two healthy subjects (19–33 years old) collected UWS by passive drooling three times daily (morning, afternoon and evening) over three consecutive days (nine timepoints). Data were analysed using linear mixed‐effects models, intraclass correlation coefficients (ICC), Bland–Altman plots and coefficients of variation (CV%).

**Results:**

Mean UWS flow rate collected over 5 min remained stable across days (0.38–0.39 mL/min), ranging from 0.14 to 0.93 mL/min. Aggregated daily means showed the highest reproducibility (ICC_3_ = 0.890 for Days 1–2), while single timepoints were less consistent (afternoon ICC_3_ = 0.524). Evening collections demonstrated the best reproducibility among individual timepoints (ICC_3_ = 0.751). Bland–Altman analysis indicated low bias and acceptable agreement across days; within‐subject variability (CVw%) was 32.24%.

**Conclusion:**

Single UWS measurements are influenced by substantial physiological variability, which may lead to diagnostic misclassification. Averaging multiple standardised collections, particularly in the evening, enhances reliability and may optimise diagnostic accuracy. This protocol supports refinement of clinical guidelines and the implementation of home‐based saliva testing for salivary gland dysfunction.

## Graphical abstract

Sixty‐two healthy participants provided unstimulated whole saliva by passive drooling for 5 min, three times daily for three consecutive days (nine samples). Single measurements showed high intra‐subject variability and limited reproducibility. Averaging repeated, standardised collections improved reliability and may enhance diagnostic accuracy.

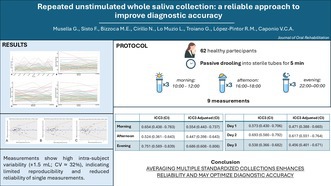

## 1 Introduction

Salivary secretion results from the coordinated function of major and minor salivary glands, producing 0.5–1.5 L per day in healthy individuals [1]. This complex process is influenced by systemic conditions, aging and medications, and its alteration may lead to hyposalivation, a measurable reduction in salivary flow, sometimes accompanied by xerostomia, the subjective feeling of dry mouth [2]. Notably, xerostomia may occur even in individuals with normal flow, while some with reduced output may be asymptomatic [3, 4]. For instance, xerostomia is frequently reported by patients with chronic oral dysaesthetic symptoms, such as those with oral dysesthetic or dysperceptive syndromes [5, 6], even when salivary flow rates are normal.

The gold standard for quantitative assessment of salivary flow is sialometry, which measures saliva production [7]. In clinical practice, both unstimulated and stimulated whole saliva are typically collected using drooling or spitting techniques over 5–15 min [8]. Recent evidence suggests that 5‐min collections are sufficient, correlating well with longer durations [9, 10]. Unstimulated whole saliva (UWS), in particular, is preferred for diagnosing hyposalivation [3, 11, 12].

However, salivary flow is subject to intra‐ and inter‐individual variability influenced by circadian rhythms, hydration and emotional or environmental factors [9, 13]. Relying on single‐timepoint measurements may lead to misclassification, especially in the absence of comorbidities or symptoms. A recent study by Varoni et al. confirmed that time of day and psychological state can significantly affect results [13].

Altered salivary secretion is frequently observed in autoimmune diseases, drug‐induced xerostomia and post‐radiation gland damage, where sialometry plays a key role in diagnosis and follow‐up [2, 10, 14]. However, despite its clinical relevance, particularly in conditions like Sjögren's disease, its use remains limited due to physiological variability and lack of standardisation. The commonly adopted cut‐off for hyposalivation (< 0.1 mL/min) may not adequately reflect true glandular function in younger or asymptomatic individuals [14], highlighting the need for a more reliable and reproducible protocol.

Despite its diagnostic potential, sialometry remains underused in clinical practice, due in part to variability in methodology and concerns about reliability. Repeated, standardised measurements could address these limitations and support broader adoption in both clinical and research settings.

Therefore, the aim of this study is to evaluate individual variability in UWS flow in a homogeneous group of healthy young adults without comorbidities and not taking medications. The primary hypothesis is that repeated UWS measurements yield a more accurate assessment of baseline gland function. The ultimate goal is to propose a standardised, reliable collection protocol for clinical implementation, enhancing the diagnostic workup of salivary disorders.

## 2 Materials and Methods

### 2.1 Study Design

This study was designed as an observational, non‐interventional, cross‐sectional investigation conducted at the Applied Research and Advanced Dental Education Services Center of the University of Foggia between November 2024 and April 2025. The study population consisted of students aged between 19 and 33 years old. Subjects of both sexes, in apparent good general health, with good oral hygiene and without smoking or alcohol consumption habits, were eligible for inclusion.

Exclusion criteria included the presence of any systemic or local disease at the time of the study, the use of any medication known to influence salivary flow (e.g., antihistamines, antidepressants/antipsychotics, antianginal agents, hormonal therapies and diuretics), and a history of oncologic conditions requiring head and neck radiotherapy, chemotherapy or immunotherapy. Specific conditions known to directly or indirectly affect salivary gland function, such as autoimmune diseases (e.g., Sjögren's disease), sarcoidosis and infectious or neoplastic diseases of the major salivary glands, were also grounds for exclusion. Participation in the study was voluntary, and participants were selected among university students who expressed willingness to take part in the study. All participants were informed of the study aims and procedures and provided written informed consent prior to enrollment. The study was conducted in accordance with the ethical principles of the Declaration of Helsinki and in compliance with the European General Data Protection Regulation (GDPR 2016/679). The study protocol was approved by the local Ethics Committee (127/CE/2023). The reporting of this observational study adhered to the STROBE (Strengthening the Reporting of Observational Studies in Epidemiology) guidelines [15]. A completed STROBE checklist is provided as Appendix S1.

### 2.2 Collection of Salivary Samples

Each participant was provided with identical sterile 15 mL conical centrifuge tubes (Falcon, Corning Inc., NY, USA), made of sterile polypropylene and graduated in 0.5 mL increments, to ensure standardisation of measurement tools. Saliva collection was performed autonomously at home after comprehensive instruction, which was provided by a member of the study team through an in‐person session, accompanied by a printed document containing detailed operational guidelines. Each participant performed three collections per day every 8 h, at predefined time windows (morning: 10:00–12:00; afternoon: 16:00–18:00; evening: 22:00–00:00), for three consecutive days, resulting in a total of nine collections per individual.

To minimise the influence of potential confounding factors and external stimuli, participants were instructed to refrain from eating, drinking (except water) and chewing gum for at least 2 h prior to collection, and to avoid smoking and performing oral hygiene procedures within 60 min prior to each collection. The use of mouthwashes or oral sprays was also discouraged during the 2 h preceding saliva collection. Collections were to be conducted in a quiet, comfortable environment free from strong olfactory stimuli, with participants seated in a relaxed position. The decision to perform saliva collection in a home setting rather than in a clinical environment was intentional, both for practical reasons and to reduce potential ‘white‐coat’ effects that could influence salivary flow measurements. To ensure adherence to the protocol timing, participants recorded the exact collection time for each sample in a structured log sheet and returned this documentation to the research team for verification. At the start of each collection, participants were instructed to swallow to clear residual saliva from the oral cavity, then started a 5‐min timer. During this period, they remained seated with their head slightly tilted forward, allowing saliva to passively drip into the sterile tube, with their mouth slightly open and their lips sealed around the tube opening, while avoiding tongue movements or voluntary swallowing. Spitting or exerting pressure to facilitate salivary flow was not permitted. At the end of the 5 min, the tube was sealed, and the collected saliva volume was initially recorded by direct reading of the graduated scale on the tube. For accurate quantification, saliva volume was determined gravimetrically. An empty tube was pre‐weighed using a precision analytical balance (±0.001 g accuracy). After collection, tubes were re‐weighed, and the net saliva weight was calculated by subtracting the empty tube weight. Volume was determined based on saliva's specific gravity of approximately 1.0 g/mL (1 g = 1 mL, or 1000 mg = 1 mL).

The measured values were recorded in an electronic database for subsequent statistical analysis. This method of salivary flow collection was selected based on previous literature suggesting that drooling provided the most reliable samples for the type of analyses conducted in this study [8].

### 2.3 Statistical Analysis

Statistical analyses were performed using R (v4.4.1). Descriptive statistics were calculated for sex and age. Normality of salivary flow distributions at each timepoint (morning, afternoon and evening) over 3 days was assessed using the Shapiro–Wilk test. As most of variables were not‐normally distributed (*p* < 0.05), non‐parametric methods were employed. Intra‐ and inter‐day differences were analysed via repeated‐measures ANOVA using linear mixed‐effects models (*lmer*) [16], including participant ID as a random intercept. The Friedman test served as a non‐parametric alternative. Post‐hoc pairwise comparisons were Bonferroni‐adjusted. Separate models were run for intra‐day, inter‐day, global (nine timepoints) and daily mean comparisons, all adjusted for age and sex. Effect sizes were expressed as generalised eta‐squared (GES), and degrees of freedom were estimated using the Kenward–Roger method. Measurement reproducibility was assessed using intraclass correlation coefficients (ICC), specifically a one‐way (ICC_1_) and a two‐way (ICC_2_) random effects single‐rater, and two‐way fixed effects single‐rater (ICC_3_). These were calculated unadjusted and adjusted for age and sex, with 95% confidence intervals (CI) estimated analytically and via bootstrapping (*n* = 500) [17]. ICCs were interpreted following Landis and Koch [18]. The Coefficient of Variation (CV%) and Standard Error of Measurement (SEM) were computed to assess variability. Bland–Altman plots, with log‐transformation when needed, evaluated agreement across days. Limits of Agreement were also expressed as a percentage of mean salivary flow.

### 2.4 Sample Size Calculation

A priori sample size estimation for the ICC was performed according to the method described by Walter et al. (1998) [19], as recommended by Koo and Li (2016) [17]. The calculation was based on an expected ICC of 0.83, reported in previous studies assessing the reliability of UWS flow measurements [8], a minimum acceptable ICC of 0.60, a 95% CI, 80% power and three repeated measures per subject. A priori sample size estimation determined that 48 subjects were required.

## 3 Results

### 3.1 Sample Characteristics

Out of 80 recruited subjects, 62 met the inclusion criteria and were included in the study. The mean age of participants was 24.26 ± 3.28 years. The sample included 38 females (61.3%) and 24 males (38.7%).

### 3.2 Mean Salivary Flow and Measurement Distribution

The mean UWS was 0.38 ± 0.15 mL/min on Day 1, 0.39 ± 0.16 mL/min on Day 2, and 0.38 ± 0.14 mL/min on Day 3, calculated from the three collections per day. The overall mean salivary flow calculated across all 3 days (total mean) was 0.39 ± 0.14 mL/min, ranging from 0.17 to 0.87 mL/min (Table 1).

**TABLE 1:** Descriptive statistics of UWS flow rate (mL/min) across the three observation days and for the overall mean.

Variable	Mean ± SD	Min	Max
Mean Day 1	0.38 ± 0.15	0.14	0.91
Mean Day 2	0.39 ± 0.16	0.17	0.93
Mean Day 3	0.38 ± 0.14	0.16	0.77
Total mean (Days 1–3)	0.39 ± 0.14	0.17	0.87

*Note:* Values are reported as mean ± standard deviation (SD), minimum and maximum.

**FIGURE 1:**
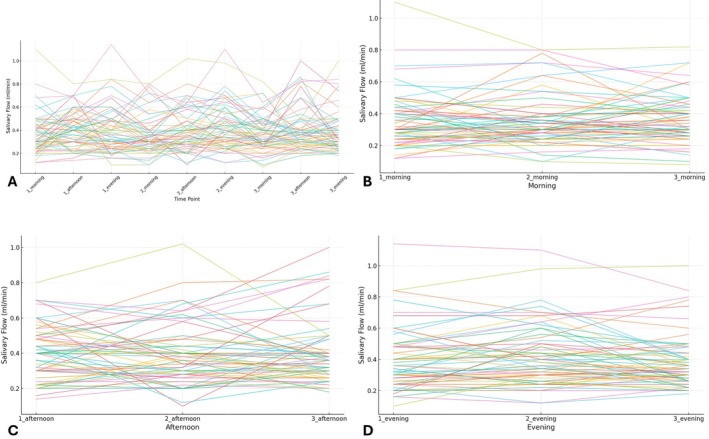
Individual trends of unstimulated salivary flow (mL/min) across different timepoints and days. (A) All timepoints across the three days (9 measurements in total); (B) Morning timepoints only (1_morning, 2_morning and 3_morning); (C) Afternoon timepoints only (1_afternoon, 2_afternoon and 3_afternoon); (D) Evening timepoints only (1_evening, 2_evening and 3_evening). Each coloured line represents an individual participant.

All individual salivary flow measurements are provided in the Appendix S2, including both raw salivary volumes (mL) collected over 5 min (Appendix S2, Table S1) and corresponding flow rates expressed as mL/min (Appendix S2, Table S2). Individual trends of unstimulated salivary flow across all timepoints and days are illustrated in Figure 1.

### 3.3 Intra‐ and Inter‐Day Differences

No significant intra‐day differences were observed on Day 1 (Friedman χ^2^ = 5.64, df = 2, *p* = 0.060) or Day 3 (Friedman χ^2^ = 4.28, df = 2, *p* = 0.118), whereas significant differences were detected on Day 2 (Friedman χ^2^ = 14.97, df = 2, *p* < 0.001). Post hoc tests for Day 2 showed a significant difference between Morning and Afternoon (*p* = 0.040, Bonferroni‐corrected) and between morning and evening (*p* < 0.001, Bonferroni‐corrected), while no difference was observed between afternoon and evening (*p* = 1.000, Bonferroni‐corrected).

No significant inter‐day differences were found when comparing the same timepoint across days (Morning: χ^2^ = 0.85, *p* = 0.652; Afternoon: χ^2^ = 0.80, *p* = 0.670; Evening: χ^2^ = 4.17, *p* = 0.124).

Taken together, these data show that while the volume of saliva collected is relatively constant throughout the day, there may be significant differences at certain time points.

### 3.4 Consistency and Reproducibility of the Measurements

Measurement consistency and reproducibility were assessed using ICC (ICC_1_; ICC_2_; ICC_3_; and ICC_3_ adjusted for age and sex), as summarised in Table 2. ICC_3_ values ranged from 0.524 to 0.890 across different scenarios. The highest consistency was observed for aggregated daily means (Mean of Day 1 and Day 2: ICC_3_ = 0.890; Mean of Days 1, 2 and 3: ICC_3_ = 0.857), whereas lower consistency was found for single‐day and single‐timepoint measurements (Day 3: ICC_3_ = 0.538; Afternoon: ICC_3_ = 0.524). Among individual time windows, evening measurements showed the highest stability (Evening: ICC_3_ = 0.751), followed by morning measurements (Morning: ICC_3_ = 0.654). The afternoon measurements had the lowest stability (Afternoon: ICC_3_ = 0.524).

**TABLE 2:** Intraclass correlation coefficients (ICC) for intra‐ and inter‐day reproducibility of unstimulated salivary flow measurements across different timepoint aggregation scenarios.

Scenario	ICC_1_ (CI)	ICC_2_ (CI)	ICC_2_ Adjusted (CI)	ICC_3_ (CI)	ICC_3_ Adjusted (CI)	Category
Mean of Day 1 and Day 2	0.890 (0.826–0.932)	0.890 (0.826–0.932)	0.843 (0.747–0.906)	0.890 (0.780–0.941)	0.850 (0.815–0.934)	Inter‐day (aggregated)
Mean of Day 1 and Day 3	0.842 (0.753–0.901)	0.842 (0.753–0.901)	0.781 (0.679–0.852)	0.842 (0.729–0.910)	0.787 (0.762–0.903)	Inter‐day (aggregated)
Mean of Day 2 and Day 3	0.838 (0.747–0.898)	0.838 (0.747–0.898)	0.783 (0.681–0.858)	0.838 (0.722–0.904)	0.786 (0.749–0.905)	Inter‐day (aggregated)
Mean of Days 1,2,3	0.857 (0.794–0.905)	0.857 (0.794–0.905)	0.800 (0.710–0.862)	0.857 (0.754–0.908)	0.809 (0.750–0.889)	Inter‐day (aggregated)
Day 1	0.563 (0.425–0.687)	0.565 (0.427–0.689)	0.443 (0.263–0.581)	0.573 (0.361–0.706)	0.471 (0.388–0.665)	Intra‐day
Day 2	0.671 (0.552–0.771)	0.674 (0.549–0.776)	0.581 (0.449–0.688)	0.693 (0.550–0.792)	0.617 (0.551–0.764)	Intra‐day
Day 3	0.533 (0.391–0.664)	0.534 (0.393–0.664)	0.447 (0.306–0.574)	0.538 (0.366–0.682)	0.456 (0.401–0.671)	Intra‐day
Morning	0.654 (0.532–0.758)	0.654 (0.532–0.758)	0.544 (0.367–0.672)	0.654 (0.438–0.783)	0.554 (0.443–0.737)	Inter‐day
Afternoon	0.524 (0.381–0.656)	0.524 (0.381–0.656)	0.444 (0.322–0.543)	0.524 (0.361–0.643)	0.447 (0.398–0.643)	Inter‐day
Evening	0.745 (0.646–0.826)	0.746 (0.646–0.827)	0.664 (0.531–0.760)	0.751 (0.589–0.839)	0.686 (0.608–0.808)	Inter‐day
All 9 timepoints	0.616 (0.524–0.710)	0.616 (0.525–0.710)	0.520 (0.408–0.598)	0.624 (0.473–0.714)	0.531 (0.514–0.644)	Inter‐day

*Note:* The table reports ICC_1_, ICC_2_, ICC_2_ adjusted for age and sex, ICC_3_, and ICC_3_ adjusted for age and sex, each with their corresponding 95% confidence intervals (CI). Adjusted ICCs were computed using linear mixed‐effects models including age and sex as fixed effects. Aggregated daily means showed higher reproducibility compared to single‐day or single‐timepoint measurements. Classification of reliability categories is based on Landis and Koch's classification [18].

When adjusting for age and sex, ICC_3_ values ranged from 0.447 to 0.850. Higher consistency remained evident for aggregated daily means (Mean of Day 1 and Day 2: ICC_3_ adjusted = 0.850, 95% CI 0.815–0.934; Mean of Days 1, 2 and 3: ICC_3_ adjusted = 0.809, 95% CI 0.750–0.889), while lower consistency was again found for single‐day or single‐timepoint measurements (Day 3: ICC_3_ adjusted = 0.456, 95% CI 0.401–0.671; Afternoon: ICC_3_ adjusted = 0.447, 95% CI 0.398–0.643). Evening measurements confirmed good consistency after adjustment (Evening: ICC_3_ adjusted = 0.686, 95% CI 0.608–0.808). Similarly, ICC_2_ values adjusted for age and sex ranged from 0.381 to 0.851 across different scenarios, with higher reliability observed for aggregated daily means (Mean of Day 1 and Day 2: ICC_2_ adjusted = 0.851 [95% CI: 0.747–0.906]; Mean of Days 1, 2 and 3: ICC_2_ adjusted = 0.800 [95% CI: 0.710–0.862]), and lower reliability for single‐day or single‐timepoint measurements (Afternoon: ICC_2_ adjusted = 0.452 [95% CI: 0.322–0.543]; Day 3: ICC_2_ adjusted = 0.447 [95% CI: 0.306–0.574]).

Measurement precision and intra‐subject variability are reported in Table 3.

**TABLE 3:** Measurement precision and intra‐subject variability of UWS flow rate (mL/min).

Index	Value
Intra‐subject standard deviation (Sw)	0.109 mL/min
Precision (1.96 × Sw)	0.213 mL/min
Reproducibility (2.77 × Sw)	0.301 mL/min
Intra‐subject standard deviation on log scale (Sw log)	0.1213
Intra‐subject coefficient of variation (CVw %)	32.24%

*Note:* Precision and reproducibility were calculated from intra‐subject standard deviation (Sw). The intra‐subject coefficient of variation (CVw %) was derived on the log‐transformed scale.

The intra‐subject standard deviation (Sw) was 0.109 mL/min, meaning that, on average, each subject’s salivary flow measurements varied by approximately 0.1 mL/min between repeated collections under the same conditions. Precision, calculated as 1.96 × Sw, was 0.213 mL/min, indicating that two measurements from the same individual are expected to differ by up to approximately ±0.21 mL/min in 95% of cases. Reproducibility, calculated as 2.77 × Sw, was 0.301 mL/min, suggesting that values collected on different days from the same subject may vary by up to approximately ±0.30 mL/min. The intra‐subject coefficient of variation (CVw %) was 32.24%, which reflects the degree of variability relative to each individual's mean; as this percentage increases, the stability of repeated measurements decreases, and single values become less representative of the subject's typical output.

Aggregated daily means showed the highest consistency and reliability across ICC models, while single‐day and single‐timepoint measures were notably less stable. Adjustments for age and sex slightly reduced ICC values but confirmed the same overall pattern, reinforcing the value of repeated measures.

### 3.5 Bland–Altman Analyses

**FIGURE 2:**
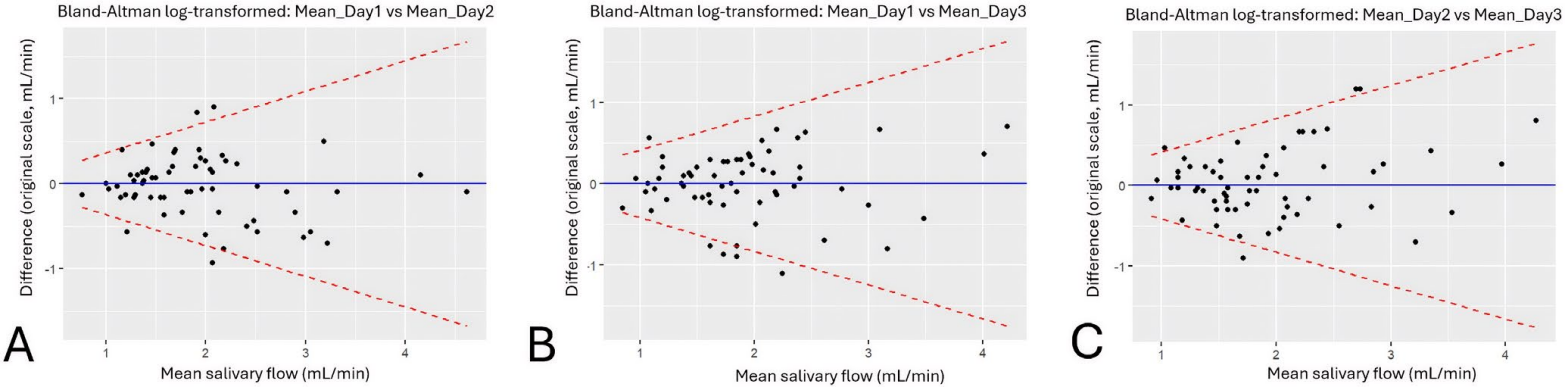
Bland–Altman plots comparing mean salivary flow measurements between days. (A) Day 1 versus Day 2; (B) Day 1 versus Day 3; (C) Day 2 versus Day 3. Analyses were performed on log‐transformed data due to heteroscedasticity, and results were back‐transformed to the original scale (mL) for clinical interpretation. The blue line represents the mean difference (bias), and the dashed red lines indicate the limits of agreement (LoA).

Agreement between salivary flow measurements across days was further evaluated through Bland–Altman analyses. Heteroscedasticity was detected in all comparisons (Kendall's *p* < 0.05), and therefore log‐transformed Bland–Altman plots were used. For the comparison between Day 1 and Day 2, the bias was −0.035 with limits of agreement (LoA) ranging from −1.674 to +1.674 (Shapiro–Wilk *p* = 0.408), corresponding to ±86.7% of the overall mean salivary flow. For Day 2 versus Day 3, the bias was +0.030 with LoA between −1.765 and +1.765 (Shapiro–Wilk *p* = 0.349), corresponding to ±91.5% of the mean. For Day 1 versus Day 3, the bias was −0.005 with LoA between −1.752 and +1.752 (Shapiro–Wilk *p* = 0.031), corresponding to ±90.8% of the mean. Overall, the Bland–Altman analyses demonstrated low bias and acceptable agreement across days, with the largest variability observed between Day 2 and Day 3. The corresponding Bland–Altman plots are shown in Figure 2.

In summary, Bland–Altman analyses showed minimal systematic bias and a good level of agreement between measurement days.

### 3.6 Linear Mixed‐Effects Models

In addition to the non‐parametric analyses, repeated‐measures ANOVA was conducted using linear mixed‐effects models to further explore intra‐day and inter‐day variations, while adjusting for age and sex as covariates and accounting for the repeated structure of the data (random intercept for subject).

For intra‐day comparisons, significant effects of timepoint were observed on Day 1 (χ^2^(2) = 6.26, *p* = 0.044) and Day 2 (χ^2^(2) = 13.62, *p* = 0.001), but not on Day 3 (χ^2^(2) = 3.63, *p* = 0.163). Post hoc contrasts confirmed that on Day 2, evening flow was significantly higher than morning flow (*p* = 0.0011 after Bonferroni correction), consistent with the results of the Friedman test.

For inter‐day comparisons conducted separately for each timepoint, no significant inter‐day differences were found for morning (χ^2^(2) = 0.43, *p* = 0.806) or afternoon measurements (χ^2^(2) = 0.24, *p* = 0.889). A trend toward significance was observed for evening measurements (χ^2^(2) = 5.09, *p* = 0.079), with post hoc contrasts approaching significance for the Day 2 versus Day 3 comparison (*p* = 0.0932).

Finally, a global repeated‐measures ANOVA across all nine timepoints (three per day) showed a significant main effect of timepoint (χ^2^(8) = 23.51, *p* = 0.0028), with some pairwise contrasts reaching significance (e.g., Day 1 Morning vs. Day 2 Evening: *p* = 0.0177). In contrast, no significant differences were found when comparing the overall daily means across the 3 days (χ^2^(2) = 0.56, *p* = 0.755), confirming the high reproducibility of average daily salivary flow.

Mixed‐effects models confirmed significant intra‐day variation, particularly on Day 2, with evening values higher than morning. No significant inter‐day differences were found for daily means, supporting the reproducibility of average salivary flow across days.

## 4 Discussion

This study provides novel insights into the intra‐individual variability and reproducibility of UWS flow in healthy young adults by employing a repeated measures design over 3 days in a home‐based setting. Our findings support the feasibility and clinical utility of using multiple assessments to capture adequate estimates of resting salivary output.

Despite standardised pre‐collection conditions and a homogeneous cohort of young healthy individuals, we observed substantial intra‐individual variability. While the average UWS flow rate remained stable across days (0.38–0.39 mL/min), single measurements fluctuated from 0.14 to 0.93 mL/min. These variations are consistent with the influence of circadian rhythms, hydration and autonomic regulation as described in prior studies [9, 14].

Importantly, 4 out of 62 participants (6.45%) showed isolated UWS values ≤ 0.1 mL/min. Specifically, two values were recorded in the morning of Day 3, one in the morning of Day 2 and one in the afternoon of Day 2. Considered the diagnosis of hyposalivation based solely on this cut‐off value, these individuals would have met one of the ACR‐EULAR criteria for salivary dysfunction in Sjögren's disease [20]. However, their mean UWS values over the 3 days were 0.23, 0.38, 0.31 and 0.25 mL/min, respectively, above the diagnostic threshold, highlighting the risk of misclassification when relying on a single time‐point and supporting averaged multi‐day assessments.

In line with our findings in healthy adults, longitudinal data in Sjögren's patients show marked within‐person variability of resting (and stimulated) flow, with individuals crossing the 0.1 mL/min cut‐off across sessions; accordingly, salivary tests used for diagnostic decisions should be obtained on several occasions to better reflect true gland function [21]. Thus, while the 0.1 mL/min threshold remains widely used, its limitations argue for protocols that integrate repeated measurements and symptom‐based tools to reduce both over‐ and under‐diagnosis. Moreover, as studies have shown, xerostomia does not always align with objective salivary flow rates, and single low measurements may not accurately capture persistent glandular hypofunction [22, 23].

Interestingly, although no consistent inter‐day differences emerged, evening measurements showed the highest reproducibility across days, with ICC_3_ of 0.751. This time window may be less influenced by diurnal variation or behavioural fluctuations and could thus represent a more reliable collection period for clinical and research use. The enhanced temporal stability observed in the evening aligns with hypotheses regarding reduced sympathetic tone, greater participant relaxation, and environmental constancy in the evening hours.

However, despite its higher reproducibility, evening saliva collection is rarely feasible in routine clinical practice. Therefore, the patient could be asked for the saliva collection from the evening before at home and bring it to the next appointment. Another option is to use the saliva from the morning, as it is done in the usual practice, since it has the second highest reproducibility.

Moreover, averaging values across days markedly improved reproducibility. The ICC for the mean of Days 1 and 2 was 0.890, classified as excellent, supporting the use of repeated, averaged values as a more robust method of baseline UWS evaluation. The variability observed across individual timepoints, while seemingly challenging for clinical application, should not discourage the use of sialometry. On the contrary, our findings highlight the importance of rigorous methodological standardisation and thoughtful interpretation. Despite moment‐to‐moment variability, group mean values remained consistent across the 3 days (ANOVA *p* = 0.755), suggesting that averaged daily flows provide a reliable estimate of baseline gland function. Therefore, the ideal way to assess salivary function would be to collect UWS over several days and not at a specific point.

Furthermore, the commonly used 5‐min drooling method was validated by our study as sufficient and practical. This supports previous work by Navazesh and Kumar (2008) [14] and Jensen et al. (2015) [12], indicating that although longer collection periods may provide marginal gains in precision, 5‐min UWS collections, when repeated and standardised, are clinically meaningful.

Our findings are consistent with Emami et al. (2022) [24], who reported that home‐based saliva collection is practical, well accepted and potentially less affected by stress. In our study, this approach contributed to strong adherence and likely minimised collection‐related anxiety, a known confounder of salivary secretion [25, 26]. Moreover, the high physiological range observed in this cohort confirms that healthy individuals may produce more than 4 mL of UWS over a 5 min collection period, consistent with early data [27].

Nevertheless, it is important to note that our cohort consisted exclusively of young adults. Given the known age‐related decline in salivary output [3, 28], our findings should not be extrapolated to older individuals or those with systemic comorbidities. The establishment of age‐specific diagnostic thresholds and reference ranges remains an important goal for future research. A logical next step will be to replicate this protocol in older or at‐risk populations to evaluate whether temporal stability, variability and optimal collection timing differ with age. Our findings reinforce the potential of saliva as a reliable diagnostic fluid when collected under standardised conditions, as highlighted in previous literature. From a clinical standpoint, optimising collection protocols and timing, especially prioritising evening assessments, may aid in the diagnosis of medication‐induced hyposalivation or salivary dysfunction related to radiation therapy or systemic diseases. Repeated sialometry could also guide the timing of therapeutic interventions or help assess the efficacy of treatments, for example salivary substitutes [3].

Despite its strengths, this study has some limitations. First, the sample consisted exclusively of young, healthy adults, which may limit the generalizability of the findings to older populations or individuals with chronic conditions. While this limits immediate extrapolation to elderly or specific population with salivary disorders, such as patients with Sjögren's disease, establishing normative variability patterns in healthy young adults provides an essential reference framework for future comparative studies in clinical populations, where medication effects, comorbidities and glandular pathology may further increase measurement variability. Secondly, saliva collection was performed solely using the drooling technique, without direct comparison to alternative methods such as spitting or suction, which may influence sample characteristics. This method was selected based on previous literature suggesting that drooling provides the most reliable and uncontaminated samples for the type of analyses conducted in this study. In the last instance, while the repeated‐measures approach improves reproducibility and reduces diagnostic error, it is more time‐consuming than single‐timepoint testing and requires high patient compliance, limiting its correct performance without the clinician's supervision.

## 5 Conclusion

Our study highlights that UWS exhibits considerable physiological variability, yet this variability can be effectively mitigated through repeated, standardised assessments. Averaging multiple measurements across days, particularly favouring evening collections, markedly enhances reproducibility and might reduce the risk of inaccurate diagnoses. These findings may have direct implications for improving diagnostic criteria for salivary gland hypofunction and call for updated clinical protocols that incorporate timepoint repetitions. It may also be useful for determining the optimal timing for diagnostic evaluation and, potentially, for chronotherapeutic administration of sialogogues in patients suffering from hyposalivation. Future research should focus on expanding these methods to older populations and on defining age‐specific reference cut‐offs to improve diagnostic precision in sialometry.

## Author Contributions

G.M.: Writing – original draft, methodology, conceptualization, formal analysis; F.S.: Data curation, investigation M.E.B.: Writing – review and editing, project administration; N.C.: Methodology, supervision; L.L.M.: Supervision, funding acquisition; G.T.: Formal analysis, validation; R.M.L.‐P.: Validation, writing – review and editing; V.C.A.C.: Writing – review and editing, supervision.

## Funding

The authors have nothing to report.

## Conflicts of Interest

The authors declare no conflicts of interest.

## Supporting information


**Appendix S1:** joor70172‐sup‐0001‐AppendixS1.docx.
**Appendix S2:** joor70172‐sup‐0002‐AppendixS2.docx.

**Table S1:** Raw data of total salivary volume (mL) collected for each participant at each timepoint. For each timepoint (morning, afternoon and evening) on Days 1, 2 and 3, unstimulated salivary volume was collected over a fixed 5‐min period. Data are reported for 62 participants (Patient_1 to Patient_62), with corresponding sex and age.
**Table S2:** Raw data of salivary flow rate measurements (expressed in mL/min) for each participant across all timepoints. For each timepoint (morning, afternoon and evening) on Days 1, 2 and 3, salivary volume was collected over a 5‐min period and converted to flow rate per minute. Data are reported for 62 participants (Patient_1 to Patient_62), with corresponding sex and age.

## Data Availability

The datasets generated and analysed during the current study are available from the corresponding author (Maria Eleonora Bizzoca, marielebizzoca@gmail.com) on request. Any material used in the study can be provided upon request in accordance with institutional policies and ethical guidelines.
