# Supplementary material for: Repeated Unstimulated Whole Saliva Collection: A Reliable Approach to Improve Diagnostic Accuracy
Source: J Oral Rehabil. 2026 Feb 25;53(6):1169–76. doi: 10.1111/joor.70172 (PMC13168835; doi:10.1111/joor.70172)
Supplement: Supplementary file 2 — Table S1: Raw data of total salivary volume (mL) collected for each participant at each timepoint. For each timepoint (morning, afternoon and evening) on Days 1, 2 and 3, unstimulated salivary volume was collected over a fixed 5‐min period. Data are reported for 62 participants (Patient_1 to Patient_62), with corresponding sex and age. Table S2: Raw data of salivary flow rate measurements (expressed in mL/min) for each participant across all timepoints. For each timepoint (morning, afternoon and evening) on Days 1, 2 and 3, salivary volume was collected over a 5‐min period and converted to flow rate per minute. Data are reported for 62 participants (Patient_1 to Patient_62), with corresponding sex and age. [file JOOR-53-1169-s001.docx]

**SUPPLEMENTARY FILE 2**

**Supplementary Table S1.** Raw data of total salivary volume (mL) collected for each participant at each timepoint. For each timepoint (Morning, Afternoon, Evening) on Days 1, 2, and 3, unstimulated salivary volume was collected over a fixed 5-minute period. Data are reported for 62 participants (Patient_1 to Patient_62), with corresponding sex and age.

| Patient_ID | 1_morning | 1_afternoon | 1_evening | 2_morning | 2_afternoon | 2_evening | 3_morning | 3_afternoon | 3_evening | Sex | Age |
| --- | --- | --- | --- | --- | --- | --- | --- | --- | --- | --- | --- |
| Patient_1 | 1.9 | 2.9 | 2.5 | 1.5 | 1.7 | 3.4 | 3.6 | 1.7 | 1.8 | F | 20 |
| Patient_2 | 1.6 | 2.4 | 1.7 | 1.5 | 1.8 | 1.2 | 1.5 | 1.6 | 1.8 | F | 25 |
| Patient_3 | 1.5 | 1.5 | 2.4 | 2.3 | 2.9 | 2.5 | 2.0 | 1.9 | 1.8 | F | 23 |
| Patient_4 | 2.0 | 2.7 | 3.5 | 2.7 | 3.0 | 3.5 | 2.2 | 2.9 | 3.3 | M | 22 |
| Patient_5 | 1.4 | 2.0 | 2.9 | 1.8 | 1.9 | 1.7 | 1.6 | 1.6 | 2.4 | M | 29 |
| Patient_6 | 1.2 | 2.2 | 1.7 | 1.5 | 1.0 | 1.2 | 1.5 | 2.4 | 1.3 | M | 21 |
| Patient_7 | 2.3 | 1.0 | 0.8 | 0.7 | 1.8 | 1.3 | 0.5 | 0.9 | 1.0 | F | 31 |
| Patient_8 | 1.0 | 1.0 | 1.2 | 1.0 | 1.2 | 1.5 | 1.0 | 1.0 | 1.2 | F | 23 |
| Patient_9 | 1.0 | 1.0 | 1.0 | 1.0 | 1.0 | 1.2 | 1.0 | 1.2 | 1.1 | F | 19 |
| Patient_10 | 1.0 | 2.0 | 1.5 | 1.5 | 1.0 | 1.8 | 1.7 | 1.8 | 1.7 | F | 27 |
| Patient_11 | 1.2 | 1.5 | 1.2 | 1.1 | 1.3 | 1.2 | 0.8 | 1.3 | 1.2 | F | 23 |
| Patient_12 | 1.1 | 1.2 | 1.0 | 1.2 | 1.2 | 1.0 | 1.3 | 1.2 | 1.0 | F | 22 |
| Patient_13 | 2.5 | 3.0 | 2.5 | 3.2 | 3.5 | 3.2 | 3.6 | 1.4 | 1.3 | M | 26 |
| Patient_14 | 1.8 | 2.0 | 2.0 | 1.9 | 2.0 | 2.1 | 2.5 | 1.8 | 2.2 | F | 23 |
| Patient_15 | 1.5 | 2.0 | 1.9 | 1.5 | 2.0 | 2.2 | 2.3 | 2.0 | 1.1 | F | 27 |
| Patient_16 | 5.5 | 4.0 | 4.2 | 4.0 | 5.1 | 4.9 | 4.1 | 2.5 | 5.0 | M | 28 |
| Patient_17 | 1.3 | 1.5 | 2.0 | 2.9 | 2.5 | 2.2 | 1.5 | 2.0 | 2.1 | F | 24 |
| Patient_18 | 1.2 | 2.4 | 1.5 | 1.4 | 2.0 | 1.3 | 1.5 | 1.8 | 1.5 | F | 21 |
| Patient_19 | 1.0 | 3.0 | 3.0 | 2.0 | 0.5 | 2.0 | 1.4 | 2.5 | 1.5 | M | 26 |
| Patient_20 | 3.4 | 3.5 | 5.7 | 3.6 | 3.2 | 5.5 | 3.2 | 4.1 | 4.2 | M | 33 |
| Patient_21 | 1.4 | 1.1 | 1.2 | 0.5 | 1.6 | 2.0 | 1.5 | 2.5 | 2.0 | F | 22 |
| Patient_22 | 2.9 | 3.5 | 3.9 | 2.7 | 3.0 | 3.1 | 2.5 | 3.4 | 2.4 | M | 21 |
| Patient_23 | 2.0 | 1.1 | 1.0 | 1.2 | 1.0 | 2.4 | 1.3 | 1.5 | 1.3 | F | 30 |
| Patient_24 | 2.1 | 2.0 | 2.0 | 1.9 | 2.0 | 2.4 | 2.1 | 1.3 | 1.8 | M | 28 |
| Patient_25 | 1.2 | 1.1 | 2.2 | 1.4 | 1.9 | 1.7 | 1.4 | 1.4 | 1.3 | F | 24 |
| Patient_26 | 1.5 | 3.0 | 2.0 | 3.9 | 2.0 | 2.1 | 1.5 | 2.0 | 2.5 | M | 24 |
| Patient_27 | 2.1 | 2.0 | 1.2 | 1.7 | 2.4 | 1.5 | 1.8 | 3.4 | 1.6 | F | 24 |
| Patient_28 | 0.6 | 0.7 | 0.8 | 0.8 | 1.1 | 0.6 | 0.9 | 1.0 | 1.1 | F | 21 |
| Patient_29 | 0.9 | 1.0 | 1.1 | 1.4 | 1.0 | 0.6 | 0.7 | 1.2 | 0.9 | F | 24 |
| Patient_30 | 3.1 | 2.0 | 2.5 | 1.5 | 1.5 | 1.9 | 2.5 | 1.6 | 1.5 | M | 26 |
| Patient_31 | 1.4 | 2.0 | 2.5 | 1.7 | 2.2 | 3.0 | 1.5 | 2.0 | 2.0 | F | 25 |
| Patient_32 | 1.4 | 1.5 | 1.0 | 1.4 | 1.4 | 1.0 | 2.5 | 2.0 | 2.0 | M | 25 |
| Patient_33 | 2.5 | 1.0 | 2.5 | 1.5 | 2.0 | 1.9 | 1.9 | 1.5 | 1.7 | F | 21 |
| Patient_34 | 1.7 | 2.7 | 4.2 | 3.2 | 4.0 | 3.5 | 2.3 | 4.1 | 3.0 | M | 26 |
| Patient_35 | 0.6 | 0.8 | 1.4 | 1.5 | 1.4 | 1.6 | 1.3 | 1.1 | 1.4 | F | 19 |
| Patient_36 | 1.1 | 1.1 | 1.2 | 1.4 | 1.4 | 1.0 | 1.0 | 1.5 | 1.5 | F | 25 |
| Patient_37 | 2.4 | 2.2 | 1.8 | 1.0 | 2.2 | 2.0 | 1.6 | 2.0 | 1.8 | F | 24 |
| Patient_38 | 1.5 | 3.0 | 3.0 | 1.9 | 2.0 | 3.7 | 2.2 | 2.7 | 2.0 | M | 23 |
| Patient_39 | 2.5 | 2.1 | 2.4 | 2.0 | 2.0 | 2.2 | 2.0 | 1.9 | 1.9 | F | 21 |
| Patient_40 | 1.0 | 2.6 | 0.5 | 0.5 | 2.1 | 1.5 | 0.4 | 1.2 | 1.5 | F | 21 |
| Patient_41 | 1.3 | 1.7 | 1.5 | 1.2 | 1.4 | 1.4 | 1.5 | 1.3 | 1.4 | F | 23 |
| Patient_42 | 1.8 | 1.5 | 1.3 | 1.2 | 1.7 | 1.5 | 1.9 | 1.4 | 1.7 | F | 23 |
| Patient_43 | 2.0 | 3.5 | 0.9 | 1.8 | 1.7 | 2.5 | 1.4 | 1.7 | 2.2 | M | 22 |
| Patient_44 | 1.5 | 2.5 | 2.4 | 1.4 | 1.9 | 2.3 | 2.4 | 2.0 | 2.4 | F | 23 |
| Patient_45 | 1.3 | 1.3 | 1.6 | 1.2 | 1.5 | 2.6 | 2.0 | 2.4 | 2.5 | M | 23 |
| Patient_46 | 0.9 | 2.2 | 1.0 | 1.2 | 0.6 | 1.1 | 2.0 | 1.2 | 1.0 | F | 21 |
| Patient_47 | 1.5 | 1.8 | 1.5 | 1.8 | 1.0 | 3.0 | 1.3 | 1.6 | 1.3 | F | 19 |
| Patient_48 | 1.2 | 1.1 | 1.3 | 1.3 | 1.5 | 1.3 | 1.4 | 1.0 | 1.0 | M | 22 |
| Patient_49 | 1.4 | 1.3 | 1.5 | 1.1 | 1.5 | 1.5 | 1.2 | 1.5 | 2.0 | M | 33 |
| Patient_50 | 1.2 | 2.2 | 3.0 | 1.5 | 2.4 | 2.0 | 1.8 | 2.1 | 2.8 | F | 25 |
| Patient_51 | 1.5 | 1.5 | 1.4 | 1.6 | 1.6 | 1.7 | 1.2 | 3.9 | 1.6 | F | 26 |
| Patient_52 | 2.2 | 1.6 | 1.8 | 1.7 | 1.3 | 1.5 | 1.6 | 1.9 | 1.3 | M | 24 |
| Patient_53 | 1.3 | 1.5 | 1.6 | 1.3 | 1.4 | 1.3 | 1.4 | 1.8 | 1.7 | F | 26 |
| Patient_54 | 2.0 | 1.6 | 1.5 | 1.7 | 3.4 | 1.8 | 3.0 | 4.3 | 1.1 | M | 22 |
| Patient_55 | 2.2 | 2.5 | 2.0 | 2.5 | 3.1 | 2.8 | 2.0 | 1.8 | 2.5 | M | 25 |
| Patient_56 | 2.4 | 2.8 | 1.8 | 2.2 | 2.2 | 1.6 | 2.1 | 2.5 | 2.5 | F | 22 |
| Patient_57 | 0.7 | 2.4 | 1.2 | 1.2 | 1.5 | 1.2 | 1.2 | 1.3 | 1.5 | F | 32 |
| Patient_58 | 2.1 | 2.5 | 2.2 | 1.9 | 3.5 | 2.7 | 3.0 | 2.0 | 3.9 | M | 25 |
| Patient_59 | 2.5 | 2.4 | 3.4 | 2.0 | 3.2 | 3.4 | 2.0 | 5.0 | 3.7 | F | 29 |
| Patient_60 | 4.0 | 3.4 | 2.4 | 4.0 | 2.9 | 3.2 | 2.9 | 4.2 | 4.0 | M | 27 |
| Patient_61 | 3.5 | 2.0 | 2.8 | 3.6 | 2.5 | 3.9 | 2.5 | 1.9 | 2.0 | M | 24 |
| Patient_62 | 1.9 | 1.8 | 1.6 | 1.5 | 1.5 | 1.7 | 1.5 | 1.5 | 1.4 | F | 21 |

**Supplementary Table S2.** Raw data of salivary flow rate measurements (expressed in ml/min) for each participant across all timepoints. For each timepoint (Morning, Afternoon, Evening) on Days 1, 2, and 3, salivary volume was collected over a 5-minute period and converted to flow rate per minute. Data are reported for 62 participants (Patient_1 to Patient_62), with corresponding sex and age.

| Patient_ID | 1_morning | 1_afternoon | 1_evening | 2_morning | 2_afternoon | 2_evening | 3_morning | 3_afternoon | 3_evening | Sex | Age |
| --- | --- | --- | --- | --- | --- | --- | --- | --- | --- | --- | --- |
| Patient_1 | 0.38 | 0.58 | 0.50 | 0.30 | 0.34 | 0.68 | 0.72 | 0.34 | 0.36 | F | 20 |
| Patient_2 | 0.32 | 0.48 | 0.34 | 0.30 | 0.36 | 0.24 | 0.30 | 0.32 | 0.36 | F | 25 |
| Patient_3 | 0.30 | 0.30 | 0.48 | 0.46 | 0.58 | 0.50 | 0.40 | 0.38 | 0.36 | F | 23 |
| Patient_4 | 0.40 | 0.54 | 0.70 | 0.54 | 0.60 | 0.70 | 0.44 | 0.58 | 0.66 | M | 22 |
| Patient_5 | 0.28 | 0.40 | 0.58 | 0.36 | 0.38 | 0.34 | 0.32 | 0.32 | 0.48 | M | 29 |
| Patient_6 | 0.24 | 0.44 | 0.34 | 0.30 | 0.20 | 0.24 | 0.30 | 0.48 | 0.26 | M | 21 |
| Patient_7 | 0.46 | 0.20 | 0.16 | 0.14 | 0.36 | 0.26 | 0.10 | 0.18 | 0.20 | F | 31 |
| Patient_8 | 0.20 | 0.20 | 0.24 | 0.20 | 0.24 | 0.30 | 0.20 | 0.20 | 0.24 | F | 23 |
| Patient_9 | 0.20 | 0.20 | 0.20 | 0.20 | 0.20 | 0.24 | 0.20 | 0.24 | 0.22 | F | 19 |
| Patient_10 | 0.20 | 0.40 | 0.30 | 0.30 | 0.20 | 0.36 | 0.34 | 0.36 | 0.34 | F | 27 |
| Patient_11 | 0.24 | 0.30 | 0.24 | 0.22 | 0.26 | 0.24 | 0.16 | 0.26 | 0.24 | F | 23 |
| Patient_12 | 0.22 | 0.24 | 0.20 | 0.24 | 0.24 | 0.20 | 0.26 | 0.24 | 0.20 | F | 22 |
| Patient_13 | 0.50 | 0.60 | 0.50 | 0.64 | 0.70 | 0.64 | 0.72 | 0.28 | 0.26 | M | 26 |
| Patient_14 | 0.36 | 0.40 | 0.40 | 0.38 | 0.40 | 0.42 | 0.50 | 0.36 | 0.44 | F | 23 |
| Patient_15 | 0.30 | 0.40 | 0.38 | 0.30 | 0.40 | 0.44 | 0.46 | 0.40 | 0.22 | F | 27 |
| Patient_16 | 1.10 | 0.80 | 0.84 | 0.80 | 1.02 | 0.98 | 0.82 | 0.50 | 1.00 | M | 28 |
| Patient_17 | 0.26 | 0.30 | 0.40 | 0.58 | 0.50 | 0.44 | 0.30 | 0.40 | 0.42 | F | 24 |
| Patient_18 | 0.24 | 0.48 | 0.30 | 0.28 | 0.40 | 0.26 | 0.30 | 0.36 | 0.30 | F | 21 |
| Patient_19 | 0.20 | 0.60 | 0.60 | 0.40 | 0.10 | 0.40 | 0.28 | 0.50 | 0.30 | M | 26 |
| Patient_20 | 0.68 | 0.70 | 1.14 | 0.72 | 0.64 | 1.10 | 0.64 | 0.82 | 0.84 | M | 33 |
| Patient_21 | 0.28 | 0.22 | 0.24 | 0.10 | 0.32 | 0.40 | 0.30 | 0.50 | 0.40 | F | 22 |
| Patient_22 | 0.58 | 0.70 | 0.78 | 0.54 | 0.60 | 0.62 | 0.50 | 0.68 | 0.48 | M | 21 |
| Patient_23 | 0.40 | 0.22 | 0.20 | 0.24 | 0.20 | 0.48 | 0.26 | 0.30 | 0.26 | F | 30 |
| Patient_24 | 0.42 | 0.40 | 0.40 | 0.38 | 0.40 | 0.48 | 0.42 | 0.26 | 0.36 | M | 28 |
| Patient_25 | 0.24 | 0.22 | 0.44 | 0.28 | 0.38 | 0.34 | 0.28 | 0.28 | 0.26 | F | 24 |
| Patient_26 | 0.30 | 0.60 | 0.40 | 0.78 | 0.40 | 0.42 | 0.30 | 0.40 | 0.50 | M | 24 |
| Patient_27 | 0.42 | 0.40 | 0.24 | 0.34 | 0.48 | 0.30 | 0.36 | 0.68 | 0.32 | F | 24 |
| Patient_28 | 0.12 | 0.14 | 0.16 | 0.16 | 0.22 | 0.12 | 0.18 | 0.20 | 0.22 | F | 21 |
| Patient_29 | 0.18 | 0.20 | 0.22 | 0.28 | 0.20 | 0.12 | 0.14 | 0.24 | 0.18 | F | 24 |
| Patient_30 | 0.62 | 0.40 | 0.50 | 0.30 | 0.30 | 0.38 | 0.50 | 0.32 | 0.30 | M | 26 |
| Patient_31 | 0.28 | 0.40 | 0.50 | 0.34 | 0.44 | 0.60 | 0.30 | 0.40 | 0.40 | F | 25 |
| Patient_32 | 0.28 | 0.30 | 0.20 | 0.28 | 0.28 | 0.20 | 0.50 | 0.40 | 0.40 | M | 25 |
| Patient_33 | 0.50 | 0.20 | 0.50 | 0.30 | 0.40 | 0.38 | 0.38 | 0.30 | 0.34 | F | 21 |
| Patient_34 | 0.34 | 0.54 | 0.84 | 0.64 | 0.80 | 0.70 | 0.46 | 0.82 | 0.60 | M | 26 |
| Patient_35 | 0.12 | 0.16 | 0.28 | 0.30 | 0.28 | 0.32 | 0.26 | 0.22 | 0.28 | F | 19 |
| Patient_36 | 0.22 | 0.22 | 0.24 | 0.28 | 0.28 | 0.20 | 0.20 | 0.30 | 0.30 | F | 25 |
| Patient_37 | 0.48 | 0.44 | 0.36 | 0.20 | 0.44 | 0.40 | 0.32 | 0.40 | 0.36 | F | 24 |
| Patient_38 | 0.30 | 0.60 | 0.60 | 0.38 | 0.40 | 0.74 | 0.44 | 0.54 | 0.40 | M | 23 |
| Patient_39 | 0.50 | 0.42 | 0.48 | 0.40 | 0.40 | 0.44 | 0.40 | 0.38 | 0.38 | F | 21 |
| Patient_40 | 0.20 | 0.52 | 0.10 | 0.10 | 0.42 | 0.30 | 0.08 | 0.24 | 0.30 | F | 21 |
| Patient_41 | 0.26 | 0.34 | 0.30 | 0.24 | 0.28 | 0.28 | 0.30 | 0.26 | 0.28 | F | 23 |
| Patient_42 | 0.36 | 0.30 | 0.26 | 0.24 | 0.34 | 0.30 | 0.38 | 0.28 | 0.34 | F | 23 |
| Patient_43 | 0.40 | 0.70 | 0.18 | 0.36 | 0.34 | 0.50 | 0.28 | 0.34 | 0.44 | M | 22 |
| Patient_44 | 0.30 | 0.50 | 0.48 | 0.28 | 0.38 | 0.46 | 0.48 | 0.40 | 0.48 | F | 23 |
| Patient_45 | 0.26 | 0.26 | 0.32 | 0.24 | 0.30 | 0.52 | 0.40 | 0.48 | 0.50 | M | 23 |
| Patient_46 | 0.18 | 0.44 | 0.20 | 0.24 | 0.12 | 0.22 | 0.40 | 0.24 | 0.20 | F | 21 |
| Patient_47 | 0.30 | 0.36 | 0.30 | 0.36 | 0.20 | 0.60 | 0.26 | 0.32 | 0.26 | F | 19 |
| Patient_48 | 0.24 | 0.22 | 0.26 | 0.26 | 0.30 | 0.26 | 0.28 | 0.20 | 0.20 | M | 22 |
| Patient_49 | 0.28 | 0.26 | 0.30 | 0.22 | 0.30 | 0.30 | 0.24 | 0.30 | 0.40 | M | 33 |
| Patient_50 | 0.24 | 0.44 | 0.60 | 0.30 | 0.48 | 0.40 | 0.36 | 0.42 | 0.56 | F | 25 |
| Patient_51 | 0.30 | 0.30 | 0.28 | 0.32 | 0.32 | 0.34 | 0.24 | 0.78 | 0.32 | F | 26 |
| Patient_52 | 0.44 | 0.32 | 0.36 | 0.34 | 0.26 | 0.30 | 0.32 | 0.38 | 0.26 | M | 24 |
| Patient_53 | 0.26 | 0.30 | 0.32 | 0.26 | 0.28 | 0.26 | 0.28 | 0.36 | 0.34 | F | 26 |
| Patient_54 | 0.40 | 0.32 | 0.30 | 0.34 | 0.68 | 0.36 | 0.60 | 0.86 | 0.22 | M | 22 |
| Patient_55 | 0.44 | 0.50 | 0.40 | 0.50 | 0.62 | 0.56 | 0.40 | 0.36 | 0.50 | M | 25 |
| Patient_56 | 0.48 | 0.56 | 0.36 | 0.44 | 0.44 | 0.32 | 0.42 | 0.50 | 0.50 | F | 22 |
| Patient_57 | 0.14 | 0.48 | 0.24 | 0.24 | 0.30 | 0.24 | 0.24 | 0.26 | 0.30 | F | 32 |
| Patient_58 | 0.42 | 0.50 | 0.44 | 0.38 | 0.70 | 0.54 | 0.60 | 0.40 | 0.78 | M | 25 |
| Patient_59 | 0.50 | 0.48 | 0.68 | 0.40 | 0.64 | 0.68 | 0.40 | 1.00 | 0.74 | F | 29 |
| Patient_60 | 0.80 | 0.68 | 0.48 | 0.80 | 0.58 | 0.64 | 0.58 | 0.84 | 0.80 | M | 27 |
| Patient_61 | 0.70 | 0.40 | 0.56 | 0.72 | 0.50 | 0.78 | 0.50 | 0.38 | 0.40 | M | 24 |
| Patient_62 | 0.38 | 0.36 | 0.32 | 0.30 | 0.30 | 0.34 | 0.30 | 0.30 | 0.28 | F | 21 |
